# Connexin 43 suppression enhances contractile force in human iPSC-derived cardiac tissues

**DOI:** 10.3389/fbioe.2025.1615953

**Published:** 2025-08-08

**Authors:** Takuma Takada, Katsuhisa Matsuura, Tatsuro Iida, Toshiharu Koike, Hidekazu Sekine, Yuhei Higashi, Tsukasa Hara, Daisuke Sasaki, Kyohei Fujita, Yuto Hinata, Junichi Yamaguchi, Tatsuya Shimizu

**Affiliations:** ^1^ Institute of Advanced Biomedical Engineering and Science, Tokyo Women’s Medical University, Tokyo, Japan; ^2^ Department of Cardiology, Tokyo Women’s Medical University, Tokyo, Japan; ^3^ Department of Pharmacology, Tokyo Women’s Medical University, Tokyo, Japan; ^4^ Tokaihit Co., Ltd., Shizuoka, Japan; ^5^ Ogino Memorial Laboratory, Nihon Kohden Corporation, Tokyo, Japan

**Keywords:** Connexin 43 (Cx43), human-induced pluripotent stem cell-derived cardiomyocytes (hiPSC-CMs), contractility, synchrony, bioengineered cardiac tissues

## Abstract

Connexin 43 (Cx43) plays a crucial role in maintaining synchronous contraction in the heart. However, it remains unclear whether Cx43 directly influences the contractile force and synchrony of entire cardiac tissues. Previously, we successfully developed human-induced pluripotent stem cell (hiPSC)-derived cardiac tissues capable of directly measuring both the contractile force of the entire tissue and cellular synchrony within it. This study aimed to evaluate whether regulating *GJA1*, the gene encoding Cx43, could enhance contractility and synchrony in these tissues. Using adeno-associated virus (AAV), we mediated *GJA1* overexpression (OE) or knockdown (shGJA1) in bioengineered hiPSC-derived cardiac tissues. Under electrical stimulation at 60 ppm, there were no significant differences in contractile force between the AAV-GJA1-OE and control tissues (0.78 ± 0.39 vs. 0.98 ± 0.43 mN, *p* = 0.32). Synchrony levels were also similar between these groups (*p* = 0.20). In contrast, shGJA1 tissues demonstrated significantly higher contractile force compared to scramble controls (1.55 ± 0.38 vs. 1.20 ± 0.15 mN, *p* = 0.039), although the difference in synchrony was not statistically significant (*p* = 0.08). RNA sequencing data revealed that a total of 37,199 genes were detected, comparing AAV6-GFP control and GJA1-OE treated hiPSC-CMs, as well as AAV6-shRNA scramble and shGJA1 treated hiPSC-CMs. We highlighted several candidate genes potentially contributing to the enhanced contractile force observed in the shGJA1 group. Furthermore, nineteen common genes were identified between the upregulation of shGJA1 compared to scramble and downregulation of GJA1-OE compared to control, which were associated with cell proliferation, transcription, contraction, and BMP signaling pathways. In conclusion, Cx43-OE did not appear to influence contractility and synchrony, meanwhile, Cx43 suppression may effectively improve contractility without impairing the synchrony in the entire cardiac tissues. Cx43 expression beyond a certain threshold may be sufficient to maintain synchronous contraction in the tissues.

## 1 Introduction

The heart’s pumping action is regulated by an electrical conduction system that coordinates synchronous contractions. Connexin 43 (Cx43) is the most abundant gap junction protein in atrial and ventricular myocytes. It forms gap junctions that reduce electrical resistance, facilitating the rapid propagation of electrical signals throughout the heart (Vermij et al., 2017). As a result, Cx43 is considered critical for maintaining synchronous contraction in the heart (Vermij et al., 2017; van Kempen et al., 1995; Fontes et al., 2012). In fact, enhanced Cx43 expression in murine-induced pluripotent stem cell (iPSC)-derived cardiomyocytes has been shown to improve intercellular coupling at the cellular level (Sottas et al., 2018). Conversely, Cx43 conditional knockout (cKO) mice experience sudden death from spontaneous ventricular arrhythmias as early as 2 months of age (Gutstein et al., 2001). Clinically, decreased Cx43 expression has been observed in failing human hearts with myofiber disarray compared to normal donor hearts (Kostin et al., 2003).

Despite these findings, it remains unclear whether Cx43 directly influences the contractile force and synchrony of entire cardiac tissues. Additionally, the threshold for Cx43 expression necessary to maintain the synchrony has not yet been established. In previous work, we successfully fabricated human iPSC-derived cardiac tissues, which allowed us to directly measure both the contractile force of the entire cardiac tissues including the basal materials, and cellular synchrony within the tissue (Takada et al., 2022; Sasaki et al., 2018). The aims of this study were to evaluate the effects of regulating *GJA1*, the gene encoding Cx43, on contractility and synchrony in human cardiac tissues, and to elucidate the underlying mechanisms.

## 2 Materials and methods

### 2.1 hiPSCs, cardiac differentiation, purification of hiPSC-CMs, and fibrin gel preparation

**FIGURE 1:**
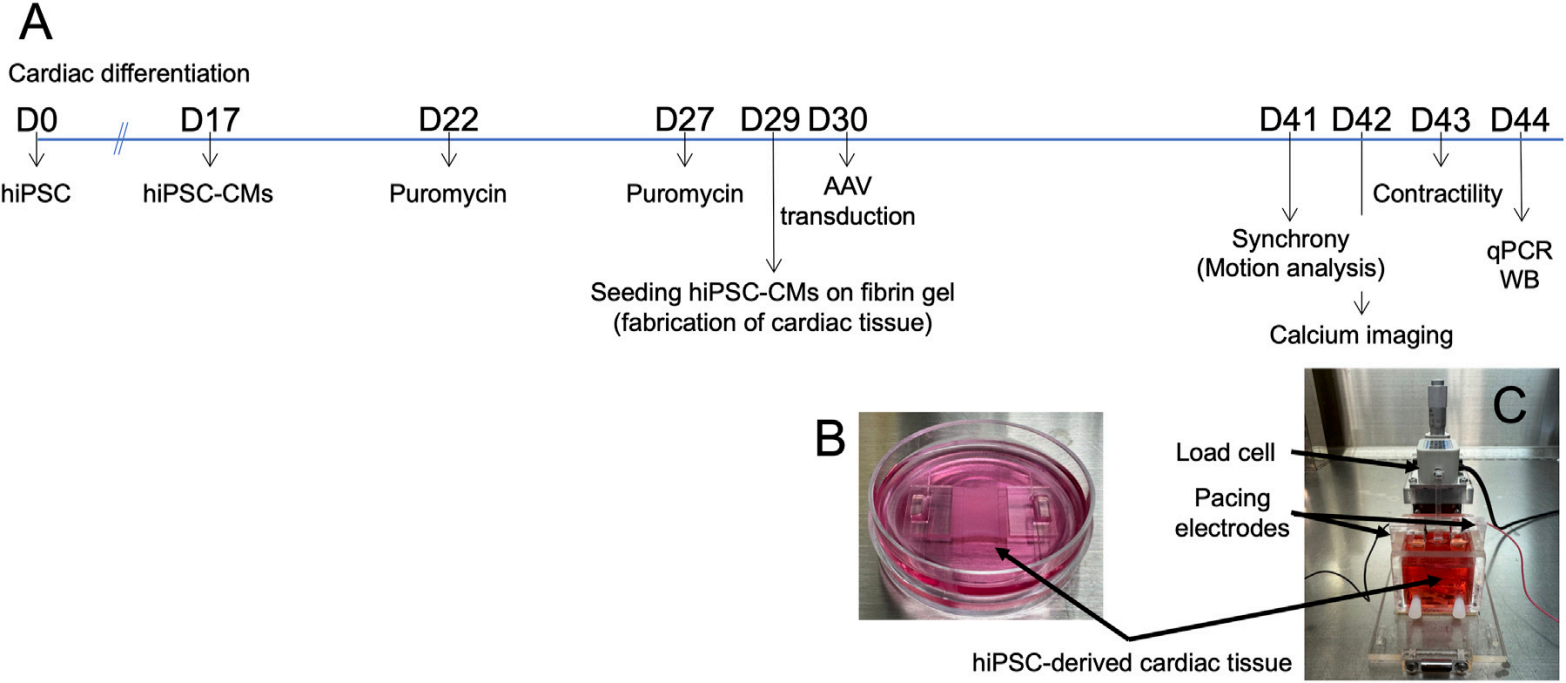
Fabrication of hiPSC-derived cardiac tissue. **(A)** Timeline of the experimental procedures. **(B)** hiPSC-derived cardiomyocytes (hiPSC-CMs) cultured on fibrin gel in a 3.5 cm dish to create cardiac tissue. **(C)** System setup for measuring contractile force. Abbreviations: AAV, adeno-associated virus; cTnT, cardiac troponin T; hiPSC-CMs, human induced pluripotent stem cell-derived cardiomyocytes; qPCR, quantitative real-time polymerase chain reaction; WB, Western blotting.

The hiPSC line 201B7 (Takahashi and Yamanaka, 2006), purchased from RIKEN (Tsukuba, Japan), expresses α-myosin heavy chain and Rex-1 promoter-driven drug-resistance genes. The cells were cultured on inactivated mouse embryonic fibroblasts (ReproCELL, Yokohama, Japan) following a previously established protocol (Matsuura et al., 2016). Cardiac differentiation was induced using a stirred bioreactor system (Bio Jr.8; Able, Tokyo, Japan) according to a published method (Matsuura et al., 2012). On day 17 of differentiation, the cell aggregates were dissociated using 0.05% trypsin/EDTA, and the cells were cultured in medium A, defined as DMEM (D6429; Sigma-Aldrich, Missouri, United States) supplemented with 10% FBS and 1% Penicillin-Streptomycin (P4333; Sigma-Aldrich), at 37°C in a humidified atmosphere with 5% CO_2_ (Figure 1A).

On day 22, the cultures were treated with 1.5 μg/mL puromycin (Sigma-Aldrich) for 23–25 h to eliminate non-cardiomyocytes that lacked puromycin resistance. The following day (day 23), the cultures were dissociated with 0.05% trypsin/EDTA and seeded onto culture dishes at a density of 1.8‒2.7 × 10^5^ cells/cm^2^. On day 27, the cultures were again treated with 1.5 μg/mL puromycin, followed by a medium change on day 28. After two rounds of puromycin selection, the purity of the hiPSC-CMs was reported to be 89% ± 9% based on cardiac troponin T expression (Takada et al., 2022).

On day 28, fibrin gel was prepared following previously described methods (Takada et al., 2022; Sasaki et al., 2018). Briefly, fibrinogen (Product# F8630; Sigma-Aldrich, Missouri, United States), thrombin (Product# T4648; Sigma-Aldrich), CaCl_2_, and lyophilized human blood coagulation factor XIII (Fibrogammin P; CSL Behring, United States) were dissolved and mixed in saline containing 0.025% polyoxyethylene (20) sorbitan monooleate (FUJIFILM Wako Pure Chemical, Osaka, Japan) at concentrations of 12.5 mg/mL, 0.5 units/mL, 2 mM, and more than 160 IU/mL, respectively. The solution was immediately poured into a silicon mold, covered with an acrylic plate, and allowed to clot, forming a fibrin gel within 30 min. After clotting, the gel was immersed in medium A supplemented with 20 μg/mL fibronectin (Corning, NY, United States) and 2.5 mg/mL aminocaproic acid (Sigma-Aldrich) for at least 2 h at 37°C. This gel was then used to seed the hiPSC-CMs.

On day 29, prior to cell seeding, the fibrin gel was fixed in a silicon mold. The purified cardiomyocyte cultures were harvested using 0.05% trypsin/EDTA, and 2.5 × 10^5^ cells were seeded onto the fibrin gel in medium A containing 1% aminocaproic acid. The following day, an adeno-associated virus (AAV6) was used to mediate *GJA1* overexpression (OE) or knockdown (shGJA1) in the bioengineered hiPSC-derived cardiac tissues. The multiplicity of infection (MOI) was set at 1.0 × 10^4^ genome copies.

On day 32, the silicon molds were carefully removed, and the hiPSC-CMs on the fibrin gel were transferred to 3.5 cm dishes (Figure 1B). The cells were immersed in medium B, defined as DMEM (low glucose, GlutaMAX™ supplement, pyruvate; Thermo Fisher Scientific, Waltham, MA, United States), supplemented with 10% FBS, 1% penicillin-streptomycin, 1% aminocaproic acid, and extracellular matrix (Matrigel^®^, Corning). The small structures holding the tissues in place were fabricated using a 3D printer (Connex3™ Objet269; Stratasys, Eden Prairie, MN, United States). From day 32 onward, the main medium was switched from medium A to medium B, with media being replaced every other day.

On day 41, microscopic videos of the cardiac tissues were recorded for motion capture analyses and the engineered cardiac tissues were placed in the contractile force measurement system (Figure 1C). Contractile properties were measured, and drug tests were performed on day 43. RNA extraction, quantitative real-time polymerase chain reaction (qPCR), and Western blotting (WB) were conducted using samples collected on day 44 (Figure 1A). The methodologies of motion capture analysis of hiPSC-CMs on fibrin gel, RNA extraction, quantitative real-time PCR, RNA sequencing, Western blotting, immunofluorescent staining, corrected field potential duration (cFPD), and simultaneous intracellular calcium imaging were described in Supplementary Material 1.

### 2.2 Contractile force measurement system

The contractile force of the cardiac tissues was assessed using a previously described measurement system (Takada et al., 2022; Sasaki et al., 2018; Figure 1C). Briefly, the system consisted of a load cell (LVS-10GA; Kyowa Electronic Instruments, Tokyo, Japan) and a culture bath constructed from acrylic plates. The engineered hiPSC-derived cardiac tissues were suspended from a sensor rod connected to the load cell using a 3D-printed hook. The lower portion of the tissue, attached to the fibrin gel, was secured with a clip at the base of the culture bath on day 41 (Figures 1B,C). 35 mL of medium C—defined as Medium 199, Hank’s (Catalog Number 12350; Thermo Fisher Scientific), supplemented with 10% FBS, 1% penicillin-streptomycin, and 1% aminocaproic acid—was added to the bath.

Contractile properties were recorded on a personal computer via an A/D converter (Power Lab 8/30; ADInstruments, Bella Vista, Australia). Electrical pacing of the cardiac tissues was performed using bipolar platinum electrodes. Biphasic pacing pulses [10 V, 10 ms pulse duration, 40–240 paces per minute (ppm)] were applied using an electronic stimulator (Nihon Kohden, SEN-3401, Tokyo, Japan). Measurements were taken at both spontaneous beat rates and during electrical pacing on day 43, capturing parameters such as contractile force, maximum contractile velocity, and relaxation velocity. The force-frequency relationship (FFR), an indicator of cardiac maturation, was also evaluated.

For drug testing, isoproterenol was added to the culture bath. A 1 mM stock solution of isoproterenol (Tokyo Chemical Industry Co., Ltd., Tokyo, Japan) was introduced into the culture medium (35 mL), achieving a final concentration of 1 μM during the contractile force measurements.

### 2.3 AAV serotype 6-mediated regulation of the GJA1 gene in hiPSC-derived cardiac tissue

We employed an AAV serotype 6 vector to mediate GJA1-OE, knockdown (shGJA1), and GCaMP expression in bioengineered hiPSC-derived cardiac tissues. The details were shown in Supplementary Material 1.

### 2.4 Statistical analysis

Continuous variables between two groups were compared using Student’s t-test. A paired t-test was applied to compare contractile force and spontaneous beating rates before and after isoproterenol administration. More than different three batches of cardiomyocytes were used in each experiment and at least three different experiments were performed. Statistical significance was defined as *p* < 0.05. All analyses were conducted using GraphPad Prism 9 (GraphPad Software Inc., CA, United States).

## 3 Results

### 3.1 GJA1 gene overexpression in hiPSC-CMs

**FIGURE 2:**
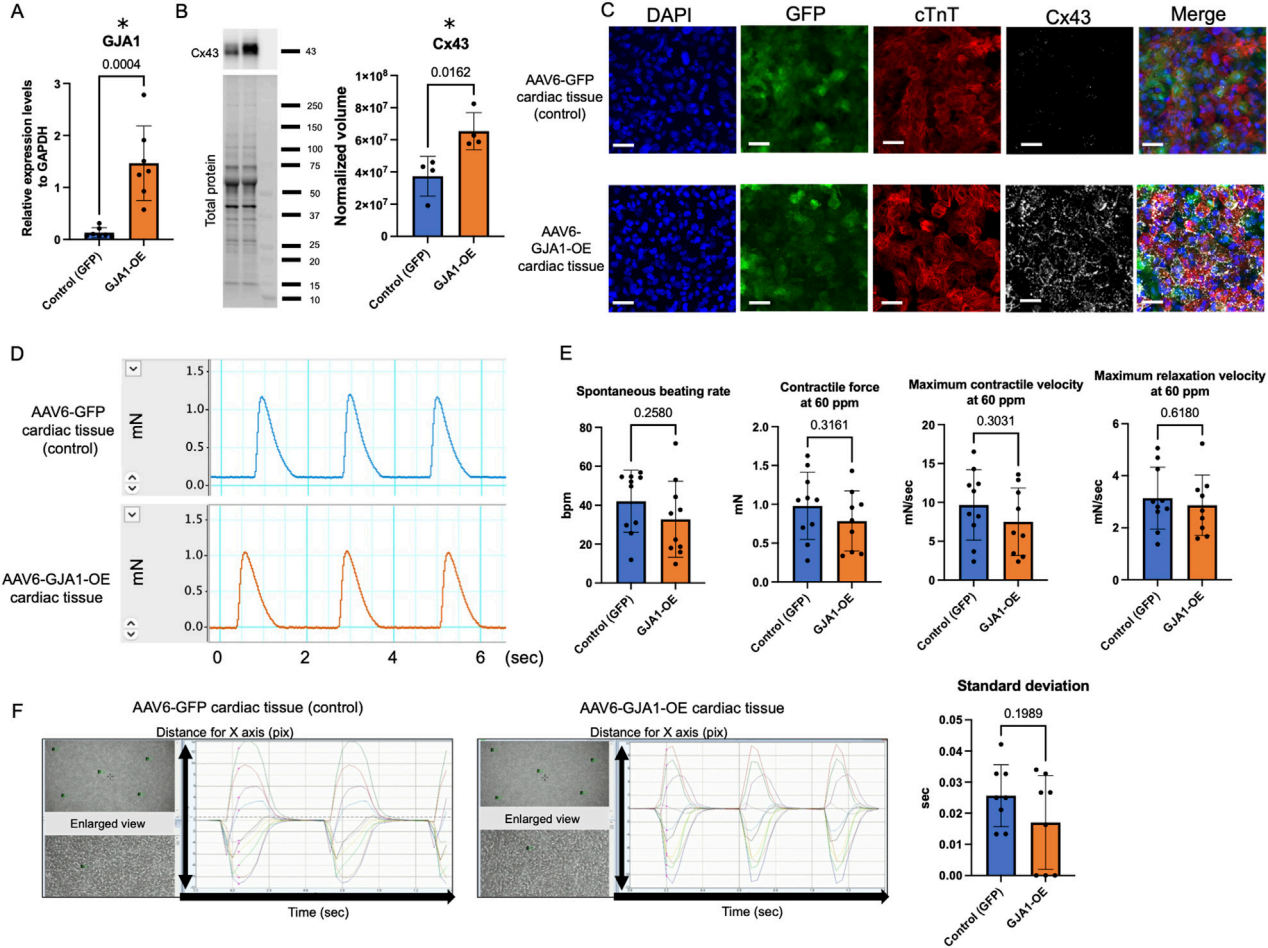
Characteristics and functions of AAV6-GJA1-OE cardiac tissues. **(A)** *GJA1* gene expression levels in AAV6-GFP and GJA1-treated hiPSC-CMs (n = 7). **(B)** Cx43 protein expression levels in AAV6-GFP and GJA1-treated hiPSC-derived cardiac tissues (n = 4). **(C)** Confocal microscopy images of AAV6-GFP and GJA1-treated cardiac tissues. Scale bar = 50 μm. **(D)** Representative contractile force data from AAV6-GFP and GJA1-treated cardiac tissues. **(E)** Spontaneous beating rate, and contractile and relaxation functions at 60 ppm in both tissue types (n = 10). **(F)** Motion capture images and analyses of AAV6-GFP and GJA1-treated tissues (n = 8). Abbreviations: AAV, adeno-associated virus; cTnT, cardiac troponin T; hiPSC, human induced pluripotent stem cells; OE, overexpression.

We first assessed the mRNA expression levels in hiPSC-CMs transduced with either AAV6-GFP (control) or AAV6-GJA1 using qPCR. As shown in Figure 2A, the mRNA expression of *GJA1* was significantly 17 times higher in AAV6-GJA1-treated hiPSC-CMs compared to the control group (*p* < 0.001). Subsequently, we generated AAV6-GJA1-OE and AAV6-GFP (control) treated hiPSC-derived cardiac tissues. Western blot analysis revealed that Cx43 protein levels were significantly 1.9 times elevated in GJA1-OE cardiac tissues compared to controls (*p* = 0.0162, Figure 2B). Confocal microscopy images (Figure 2C; Supplementary Figure S1) confirmed these findings, showing a higher expression of Cx43 in the GJA1-OE cardiac tissue compared to the control. In the GJA1-OE group, some areas of Cx43 appeared aggregated (Supplementary Figure S1). There were no significant differences observed in the expression of *MYL2*, *TNNI3*, calcium-handling genes, *GJA5*, *GJC1*, or cardiomyocyte maturation-related genes between the two groups (Supplementary Figure S2). When the expression level in normal human adult hearts was set to 1, the levels in GJA1-OE group were higher than 1 without 95% confidence interval (CI) crossing 1, indicating higher expression in the AAV6-GJA1-treated hiPSC-CMs compared to that in adult [mean and 95% CI: GFP (control); 2.10 and 0.796–3.414, GJA1-OE; 23.5 and 13.7–33.4, Supplementary Figure S3].

### 3.2 Contractile properties in AAV6-GJA1-OE cardiac tissues

We next assessed the impact of *GJA1*-OE on the contractile function of cardiac tissues on day 43 (Figures 2D,E). Figure 2D shows a representative image of the contractile force trace. There were no significant differences in the spontaneous beating rate, contractile force, maximum contraction and relaxation velocities at both 60 ppm and spontaneous beating rate between AAV6-GFP and AAV6-GJA1-OE group (Figure 2D; Supplementary Figure S4A). The FFR was negative, indicating the tissues were immature (Supplementary Figure S4B).

Next, we performed an isoproterenol treatment to the tissues. The treatment significantly increased both the spontaneous beating rate and contractile force in both groups. In the control group, the spontaneous beating rate increased from 42 ± 16 beats per minute (BPM) to 62 ± 12 BPM, and the contractile force increased from 0.98 ± 0.42 mN to 1.1 ± 0.53 mN (paired *t*-test: *p* < 0.001 and *p* = 0.008, respectively). Similarly, in the GJA1-OE group, the spontaneous beating rate rose from 33 ± 20 BPM to 59 ± 20 BPM, and the contractile force increased from 0.75 ± 0.43 mN to 0.84 ± 0.48 mN (paired *t*-test: *p* < 0.001 and *p* = 0.004, respectively). However, no significant differences were observed between the groups in terms of these parameters or in the contraction and relaxation velocities, even under electrical stimulation at 100 BPM (Supplementary Figures S4C,D).

We also evaluated the cFPD in AAV6-GFP and GJA1-OE-treated hiPSC-CMs. Both groups showed identical cFPD values, both before and after isoproterenol treatment (both *p* = 0.32, Supplementary Figure S4E). Additionally, we assessed the contractile synchrony in the cardiac tissues using motion analysis. As illustrated in Figure 2F, the timing of peak contraction was measured at five designated points, and the standard deviation of the time from the beginning of the measurement to the first peak contraction, which reflects the variation in contraction among individual cardiomyocytes, showed no significant difference between the two groups (*p* = 0.20). These findings suggest that overexpression of Cx43 does not significantly influence either contractility or synchrony in the engineered cardiac tissues.

### 3.3 GJA1 gene knockdown in hiPSC-CMs

**FIGURE 3:**
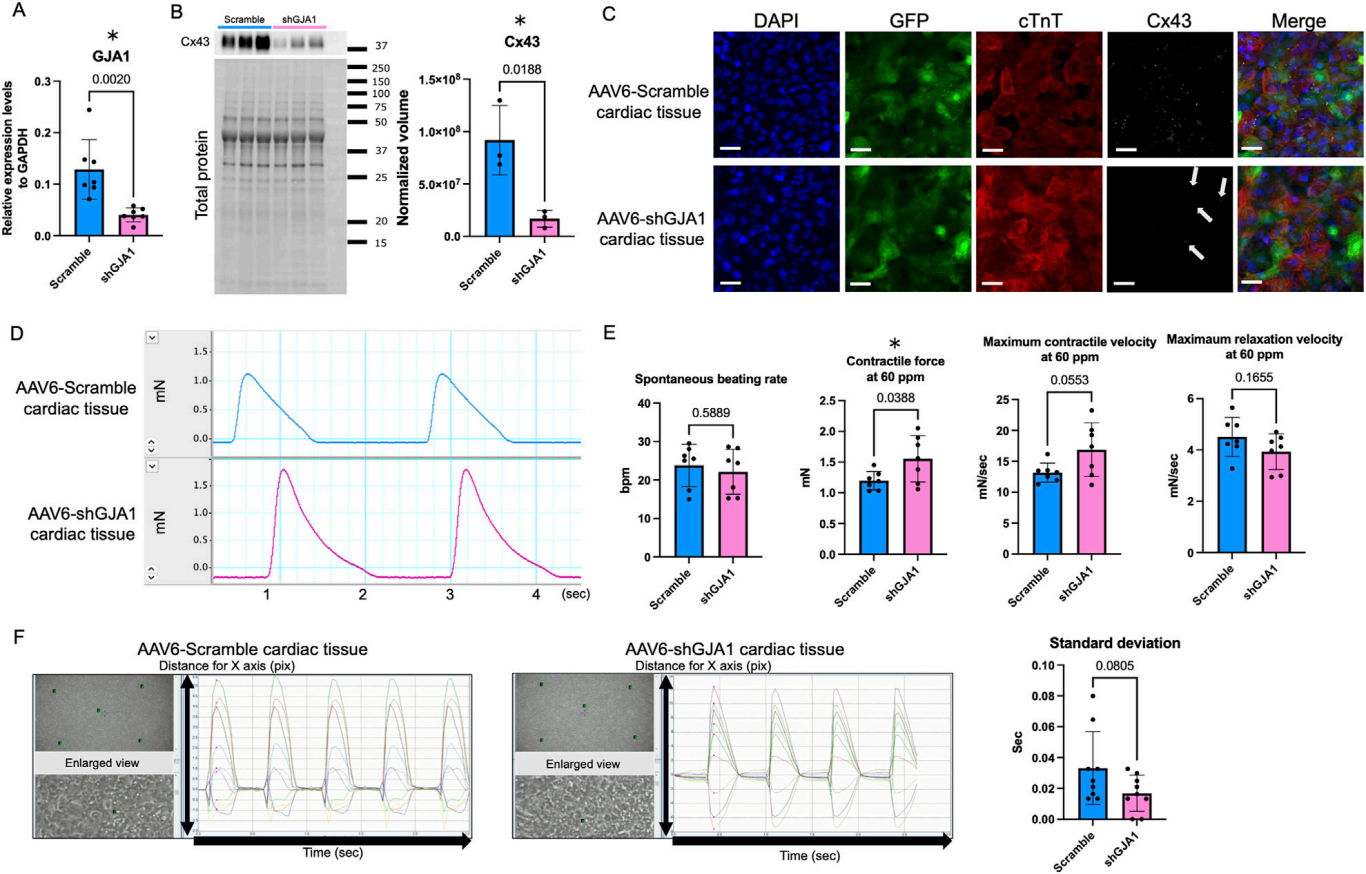
Characteristics and functions of AAV6-shGJA1 cardiac tissues. **(A)** *GJA1* gene expression levels in AAV6-Scramble and shGJA1-treated hiPSC-CMs (n = 7). **(B)** Cx43 protein expression levels in AAV6-Scramble and shGJA1-treated tissues (n = 3). **(C)** Confocal microscopy images of AAV6-Scramble and shGJA1-treated tissues. Scale bar = 50 μm. **(D)** Representative contractile force data from AAV6-Scramble and shGJA1-treated tissues. **(E)** Spontaneous beating rate, and contractile and relaxation functions at 60 ppm (n = 7). **(F)** Motion capture images and analyses of AAV6-Scramble and shGJA1-treated tissues (n = 9). Abbreviations: AAV, adeno-associated virus; cTnT, cardiac troponin T; hiPSC, human induced pluripotent stem cells; ISP, isoproterenol; ppm, pacing per minute.

To achieve *GJA1* knockdown in hiPSC-CMs, we utilized AAV6 to mediate the delivery of shGJA1. As shown in Figure 3A, mRNA expression levels of *GJA1* were significantly 0.35 times reduced in AAV6-shGJA1-treated hiPSC-CMs compared to those treated with AAV6-scramble shRNA (*p* = 0.002). No significant differences were found between the two groups regarding the expression of *MYL2*, *TNNI3*, calcium-handling genes, *GJA5*, *GJC1*, or genes related to cardiomyocyte maturation (Supplementary Figure S5). Then, the GJA1 expression levels in shGJA1 group were low than 1 without 95% CI crossing 1, indicating lower expression in the AAV6-shGJA1-treated hiPSC-CMs compared to that in adult (mean and 95% CI: scramble; 2.07 and 1.27–2.86, shGJA1; 0.652 and 0.467–0.837, Supplementary Figure S3).

Subsequently, we fabricated hiPSC-derived cardiac tissues treated with AAV6-shGJA1 and AAV6-scramble shRNA. Western blot analysis revealed that the protein levels of Cx43 were significantly 0.18 times lower in the shGJA1-treated cardiac tissues compared to the scramble-treated group (*p* = 0.0188, Figure 3B). Confocal microscopy images (Figure 3C; Supplementary Figure S1) confirmed these findings, showing a reduced presence of Cx43 in the shGJA1 group compared to the scramble group, although some Cx43 expression was still detectable in the shGJA1 tissues. Interestingly, despite the knockdown, the AAV6-shGJA1-treated hiPSC-cardiac tissue exhibited spontaneous contraction with synchronizing adjacent cardiomyocytes, similar to the AAV6-scramble shRNA-treated cardiac tissue on day 41 (Supplementary Videos S1, S2). Additionally, synchronized spontaneous intracellular calcium elevations were observed between adjacent cardiomyocytes in both treatment groups on day 42 (Supplementary Videos S3, S4; Supplementary Figure S6).

### 3.4 Contractile properties in AAV6-shGJA1 treated cardiac tissues

We next evaluated the impact of shGJA1 on the contractile function of cardiac tissues on day 43. Figure 3D shows a representative contractile force trace. Although the spontaneous beating rate was similar between the two groups, the contractile force and maximum contractile velocity of the AAV6-shGJA1 treated cardiac tissues at 60 ppm were significantly higher than those of the AAV6-scramble shRNA treated tissues (1.55 ± 0.38 vs. 1.20 ± 0.15 mN, *p* = 0.039, Figure 3E). Similar results were observed at the spontaneous beating rate; 1.66 ± 0.46 vs. 1.25 ± 0.17 mN, *p* = 0.047, Supplementary Figure S7A). FFR in the tissues was also negative (Supplementary Figure S7B)

Following isoproterenol treatment, the spontaneous beating rate and contractile force in the scramble group increased from 24 ± 5.5 BPM and 1.25 ± 0.17 mN to 55 ± 7.0 BPM and 1.40 ± 0.20 mN (both *p* < 0.001, paired *t*-test). Similarly, in the shGJA1 group, the spontaneous beating rate and contractile force increased from 22 ± 5.8 BPM and 1.66 ± 0.46 mN to 51 ± 7.4 BPM and 1.84 ± 0.48 mN (both *p* < 0.001, paired *t*-test). There were no significant differences in the spontaneous beating rate between the two groups after isoproterenol treatment (*p* = 0.24), but the contractile force in the AAV6-shGJA1 treated cardiac tissues was significantly higher than in the AAV6-scramble shRNA tissues (*p* = 0.045, Supplementary Figure S7C). Similar trends were observed under electrical stimulation at 100 ppm in both groups (Supplementary Figure S7D).

Next, we evaluated the cFPD in AAV6-scramble shRNA and shGJA1 treated hiPSC-CMs. The cFPD, both before and after isoproterenol treatment, was comparable between the two groups (*p* = 0.40 and *p* = 0.17, respectively, Supplementary Figure S7E). Furthermore, as shown in Figure 3F, motion analysis revealed the degree of contractile synchrony in the cardiac tissues. The standard deviation of the time from the start of measurement to the first peak contraction at five designated points was not significantly different between the two groups (*p* = 0.081). These findings suggest that suppression of Cx43 may enhance contractility but does not significantly affect synchrony in the cardiac tissues.

### 3.5 Mechanistic insights into enhanced contractile force in AAV6-shGJA1 cardiac tissues and downstream effects of GJA1 gene regulation

**FIGURE 4:**
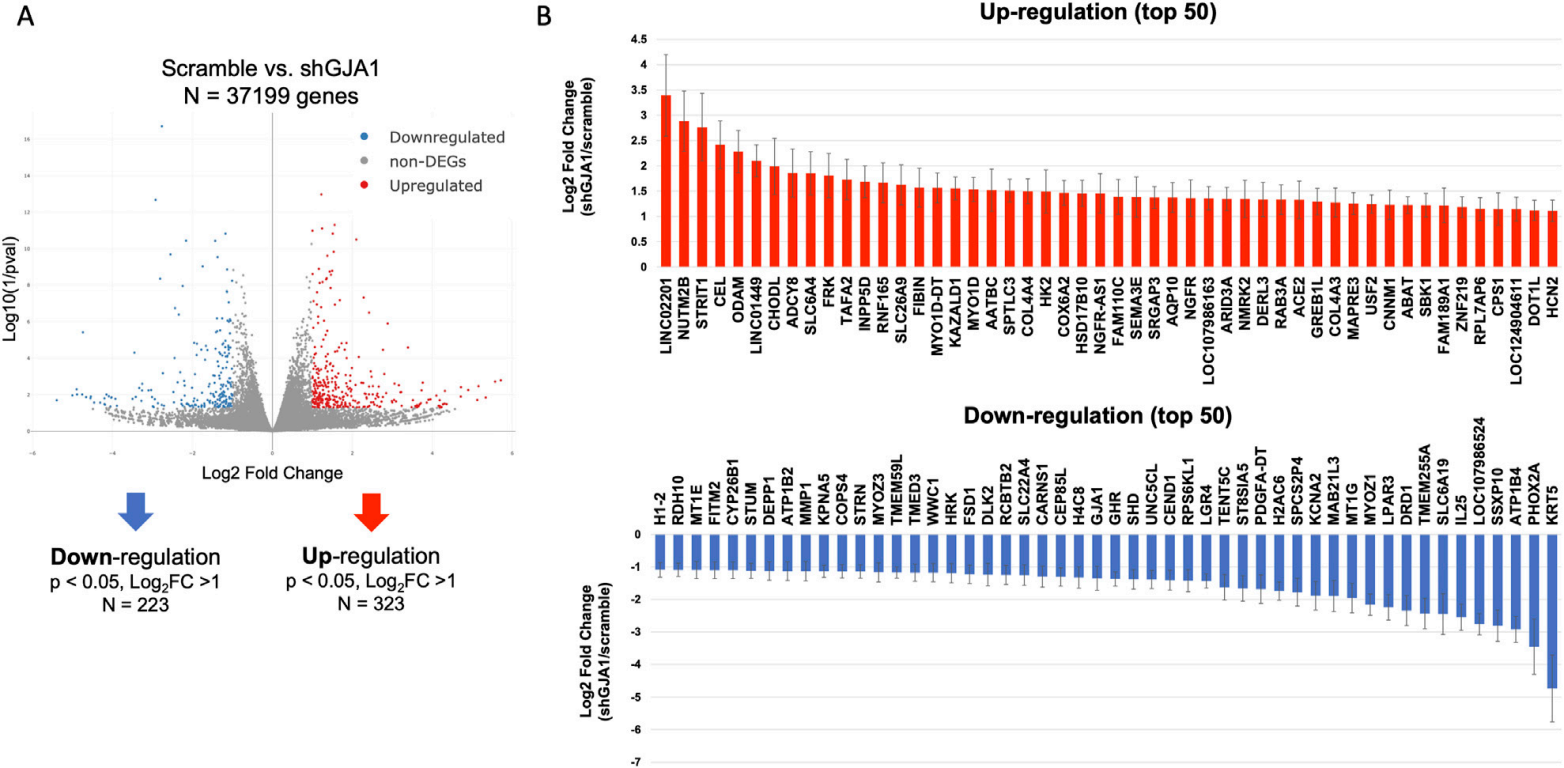
Transcriptomic impact of GJA1 suppression. **(A)** RNA sequencing and volcano plot analysis (n = 3). **(B)** DEGs involved in shGJA1, represented as the log_2_ fold change relative to scramble Error bar indicates standard error. DEG, differentially expressed gene.

To further investigate the underlying mechanisms of enhanced contractile force in AAV6-shGJA1 cardiac tissues, RNA sequencing was performed. A total of 37,199 genes were detected in the comparison between AAV6-shRNA scramble and shGJA1-treated hiPSC-CMs. Among these, 323 genes were significantly upregulated and 223 genes were significantly downregulated in the shGJA1 group compared to the scramble control. The heat maps of the differentially expressed genes were shown in Supplementary Figure S8. Then, the gene ontology analysis showed that the negative regulation of cell differentiation, muscle myosin complex, chromatin, and etc., were related to the suppression of GJA1 (Supplementary Figure S9). We additionally showed top of 50 upregulated and downregulated genes among the significantly up or downregulated genes. These genes were considered potential candidates associated with the mechanisms underlying the improvement in contractile force (Figure 4).

Next, we further assessed the downstream effects of GJA1 gene regulation, as its impact remains unclear. A total of 37,199 genes were also detected in the comparison between AAV6-GFP and GJA1-OE treated hiPSC-CMs. Of those, 211 genes were significantly downregulated in the GJA1-OE group compared to the control. The heat maps of the differentially expressed genes were shown in Supplementary Figure S8. We combined the data on genes upregulated in shGJA1 compared to scramble with those downregulated in GJA1-OE compared to control, and identified nineteen common genes that were regulated in opposite directions (Supplementary Figure S10A). Conversely, by combining the data on genes downregulated in shGJA1 compared to scramble and upregulated in GJA1-OE compared to control, we identified two genes; GJA1 and NPIPA9. NIPIPA9 has no biological process term. Among these, the common 19 genes were associated with cell proliferation, transcription, contraction, and BMP signaling pathways (Supplementary Figure S10B). Of these, the transforming growth factor alpha (TGFA) gene showed the highest upregulation in shGJA1-treated hiPSC-CMs compared to the AAV6-scramble group (log_2_FoldChange = 3.2, Supplementary Figure S11). Moreover, expression of the ERBB1 and ERBB4 genes, both TGFA- receptors, was detected in hiPSC-CMs (Supplementary Figure S5). In contrast, immunoglobulin kappa constant (IGKC) was the most downregulated in GJA1-OE-treated hiPSC-CMs compared to the AAV6-control group (log_2_FoldChange = –5.0).

## 4 Discussion

In this study, we used AAV6 to regulate *GJA1* gene expression in bioengineered hiPSC-derived cardiac tissues and assessed the effects on tissue function and underlying mechanisms. The key findings of the study were: 1) Overexpression of Cx43 did not significantly influence contractile properties or synchrony, and 2) suppression of Cx43 enhanced contractile force without impairing synchrony. These results suggest novel functional roles for Cx43.

In the heart, three major gap junction proteins are present in conductive and working myocardial cells: Cx40, Cx43, and Cx45. Cx40 is primarily expressed in atrial myocytes and the cardiac conduction system, while Cx43 is the most abundant, extensively expressed in atrial and ventricular myocytes, with lower expression in parts of the ventricular conduction system. Cx45 is mainly localized to the sinoatrial node, atrioventricular node, and the conduction system (van Kempen et al., 1995; Fontes et al., 2012). The hiPSC-CMs used in our study developed a myosin light chain 2v (MLC2v)-positive ventricular phenotype, with the *GJA1* gene (encoding Cx43) expressed at significantly higher levels than the *GJC1* and *GJA5* genes (Takada et al., 2022; Sasaki et al., 2018). Consequently, we focused our investigation on Cx43.

Although the iPSC-CMs exhibited lower *GJA1* expression compared to neonatal mouse cardiomyocytes (Marcu et al., 2015), intercalated disc-like structures were still observed between neighboring iPSC-CMs, even with the reduced levels of Cx43 (Kiss et al., 2022). In our study, tissues treated with GJA1-OE showed some Cx43 was aggregated, though Cx43 was also distributed circumferentially, with endogenous Cx43 detected (Figure 2C; Supplementary Figure S1). Previous research has reported slower conduction velocity in murine stem cell-derived cardiomyocytes compared to primary cardiomyocytes (Kucera et al., 2015). Sottas et al. (2018) found that increased Cx43 expression enhanced gap junction formation and significantly improved the spatiotemporal characteristics of signal propagation in murine iPSC-CMs. However, these studies focused on cellular-level analysis, and the effect of basal materials on the cardiomyocyte contraction may be of concern because the cardiomyocytes are fixed on the experimental polystyrene dish with high Young’s modulus (Kurotsu et al., 2020). We believe that it is also important to assess the function of the entire cardiac tissue because it indicates the cellular functional integration. Using our original methods with silicon mold and the fibrin gel which is comparable to Young’s modulus of native human heart, the cardiac tissue can be fixed to not only the contractile measurement system but also a 3.5 cm dish without the bottom sticking on the dish (Takada et al., 2022). Utilizing this system, the current study investigated how Cx43 regulation affected contractility and synchrony at entire human iPSC-derived cardiac tissues.

Next, we explore the underlying mechanisms behind the improved contractile force observed in AAV6-shGJA1 cardiac tissues. Actually, negative regulation of cell differentiation, muscle myosin complex, chromatin, and etc., were documented in the gene ontology analyses. We further performed gene selection among those that were upregulated or downregulated in shGJA1 compared to the scramble control. Among the top 50 upregulated genes, *LINC02201*, *ODAM*, *LINC01449*, *KAZALD1, SEMA3E, ARID3A, DERL3, RAB3A, and SBK1* have been reported to promote cell proliferation (Wu et al., 2020; Hou et al., 2023; Wang et al., 2013; Shen et al., 2022; Angelis et al., 2024; Shibata et al., 2017; Kim et al., 2014; Chen et al., 2023). Additionally, *ADCY8* and *COX6A2* have been associated with enhanced cardiac contractile force (Tarasov et al., 2022; Jiang et al., 2023). *DERL3* and *NMRK2* have been reported to play potentially protective roles in the failing heart (Belmont et al., 2010; Diguet et al., 2018). Conversely, among the top 50 downregulated genes, *PHOX2A*, *SLC6A19*, *CEND1, CEP85L, HRK, WWC1, KPNA5, MMP1, and ATP1B2* have been reported to inhibit cell proliferation (Wang et al., 2016; Chang et al., 2025; Liu et al., 2024; Lu et al., 2020; Wang et al., 2022; Chen, 2022; Gao et al., 2023; Du et al., 2022; Kong et al., 2021), while *DRD1*, *DEPP1, and STRN* has been negatively correlated with cardiac contraction (Nakamura et al., 2022; Wyant et al., 2024; Chacar et al., 2025). Although these findings are consistent with our results, further investigation is warranted to elucidate the underlying mechanisms linking cell proliferation and enhanced contractile force through Cx43 suppression.

Regarding the regulation of *GJA1* gene, RNA sequencing revealed that the regulation of Cx43 was associated with key processes such as cell proliferation, transcription, contraction, and BMP signaling. *TGFA* gene showed the highest upregulation in shGJA1-treated hiPSC-CMs compared to the AAV6-scramble group. Meanwhile, *IGKC* was the most downregulated in GJA1-OE-treated hiPSC-CMs compared to the AAV6-control group. Among the 19 commonly identified genes, *IGKC*, *HLA-DQB1-AS1*, and *C11orf21* did not have any biological process term. Of the rest, we focused on *TGFA* gene. Previous studies have shown that *TGFA* is linked to ERBB signaling, cell survival, and growth (Hua et al., 2016; Xu et al., 2020), while Cx43 has been reported to inhibit cancer cell growth through cell-cell communication or protein-protein interactions (Mehta et al., 1999; Ionta et al., 2009). Overexpression of Cx43 has also been shown to suppress cell cycle progression in HuH-7 cells (Hino et al., 2015). Various mechanisms have been proposed for Cx43-mediated inhibition of cell proliferation (Graham et al., 2018). Another previous reports showed that the injured heart showed de-differentiation and proliferation, especially in immature cells (Ogawa et al., 2021; Yuan and Braun, 2017; Porrello et al., 2011). Although we confirmed the expression of ERBB1 and ERBB4 gene, both TGFA- receptors, in hiPSC-CMs, the detailed interactions between *TGFA* and Cx43 remain unclear. However, we believe that the identification of 19 genes showing reciprocal expression changes provides meaningful insights into downstream pathways affected by GJA1 because the effects of regulating *GJA1*, the gene encoding Cx43, on contractility and synchrony in human cardiac tissues, as well as the underlying mechanisms, remain unclear.

Our findings might be limited in developmentally immature human iPSC-derived cardiac tissues because a previous report showed the downregulation of Cx43 *in vivo* studies did not have any discernible effect on contractile heart function (Gutstein et al., 2001). Relative mRNA expression levels of Cx43 in murin ESC- and iPSC-CMs were approximately 1/10 compared to the neonatal Cx43 expression (Marcu et al., 2015). Actually, the contractile force of native human cardiac tissue was 16–44 mN/mm^2^ (Mashali et al., 2021; Hasenfuss et al., 1991), meanwhile, our fabricated human iPSC-derived cardiac tissues showed 1.7–3.3 mN/mm with negative FFR and lacked other types of cells (Takada et al., 2022; Sasaki et al., 2018). In the current study, SCN5A gene expression did not change after overexpression or downregulation of Cx43, but the NaV1.5 channels localization might be changed (Sottas et al., 2018). However, we have reported hiPSC-derived cardiac tissues shows physiologically similar characteristics to native human heart. For instance, the contraction and beating ratio increased in response to the isoproterenol in the tissues. It is similar to those in native human heart in clinical practice. We further have reported the contraction and beating rate in response to drugs such as propranolol, verapamil, thapsigargin, ryanodine, blebbistatin, ivabradine, and omecamtiv mecarbil. Although some gene expression levels were low compared with human primary heart, the characteristics of the drug response in hiPSC-derived cardiac tissues were also similar to clinical findings in human heart (Hinata et al., 2024). Additionally, the effect of adrenaline administration on the contractile force and beating rate of hiPSC-derived cardiac tissues was examined. Both the contractile force and the beating rate significantly increased within a few minutes. Furthermore, when we stretched the tissues by 5% up to 20%, the contractile force increased, suggesting that it was physiologically relevant to actual human cardiac tissue, known as Frank-Starling mechanism (Sasaki et al., 2018).

To summarize the results of this study, the gene expression of *GJA1* and protein expression of Cx43 were 17 and 1.9 times higher, respectively, in AAV6-GJA1-OE-treated hiPSC-derived cardiac tissues compared to controls. However, no significant differences in contractility or synchrony were observed. In contrast, in AAV6-shGJA1-treated tissues, *GJA1* gene expression and Cx43 protein levels were reduced to 0.35 and 0.18 times, respectively, compared to the scramble group. Contractility was significantly improved in the shGJA1 group compared to the scramble group, but synchrony did not differ significantly between the two groups. Previous reports showed that the GJA1 gene expression levels in some hiPSC-CMs or hiPSC-derived cardiac tissues were similar or higher compared to that of human adult heart (Besser et al., 2018; Kodama et al., 2019). The GJA1 gene expression levels in control and scramble were consistent with those reports, but another report showed the opposite results (Ronaldson-Bouchard et al., 2018). Although the polarization of intercalated discs, including Cx43, has been associated with the maturation of hiPSC-CMs (Karbassi et al., 2020), such polarization was not clearly observed in either the control or scramble group in the current study. The direct relationship between GJA1 gene expression and the maturation state of hiPSC-CMs does not appear to be fully established. Next, we showed relative values of GJA1 overexpression or shGJA1 to control or scramble, respectively, but a clear indication of the normal Cx43 level was limited. However, both hiPSC-derived cardiac tissues with low (mean 0.652 times; shGJA1) and high GJA1 gene expression levels (mean 23.5 times; GJA1-OE), compared to adult normal human heart, exhibited synchronous contraction. It should be noted that AAV-mediated overexpression and shRNA-mediated knockdown of endogenous GJA1 (Cx43) are physiologically distinct approaches. We have compared GJA1-OE only with its corresponding control, and shRNA-mediated knockdown (shGJA1) with the scramble RNA control group. Accordingly, we have not conducted any direct comparisons between the GJA1-OE and shGJA1 groups.

Previous studies have shown that Cx43 knock-out mice embryos die at birth due to a failure in pulmonary gas exchange caused by swelling and blockage of the right ventricular outflow tract (Reaume et al., 1995). Moreover, mice with a cardiac-specific loss of Cx43 exhibited normal heart structure and contractile function but developed sudden cardiac death due to spontaneous ventricular arrhythmias by 2 months of age (Gutstein et al., 2001). In our study, although Cx43 expression was reduced, it was not completely abolished, and residual Cx43 may have contributed to maintaining synchrony. Interestingly, Wang et al. demonstrated that gap junctional and ephaptic coupling synergistically but redundantly excited myocytes coupled to fibroblasts. They further suggested that connexin-dependent gap junction coupling was not essential for fibroblast-myocyte electrical coupling, as ephaptic coupling mediated myocyte excitation at low gap junction conductance (Wang et al., 2023). In our study, electrical coupling between myocytes was observed in calcium imaging, even in the population with low Cx43 expression levels. Based on these findings, we propose that the expression level of Cx43, if present above a certain threshold, may be sufficient to maintain synchronous contraction at the tissue level.

Finally, as a clinical implication, in failing human hearts with systolic dysfunction, both myofiber disarray and reduced Cx43 expression were observed compared to healthy donor hearts (Kostin et al., 2003). In light of our current results, we speculate that the suppression of Cx43, which improves contractile force without compromising synchrony, may represent a compensatory mechanism in the context of systolic dysfunction. Further investigation is needed to elucidate the role of Cx43 in failing heart.

## 5 Conclusion

Overexpression of Cx43 does not appear to significantly impact either contractility or synchrony in hiPSC-derived cardiac tissues. In contrast, Cx43 suppression may enhance contractility without compromising tissue-wide synchrony. Furthermore, Cx43 expression levels above a certain threshold may be sufficient to maintain synchronous contraction in the entire cardiac tissues.

## Data Availability

The original contributions presented in the study are publicly available. This data can be found here: https://www.ncbi.nlm.nih.gov/geo/query/acc.cgi?acc=GSE304132.

## Supplementary material

SUPPLEMENTARY VIDEO 1 AAV6-scramble shRNA-treated hiPSC-derived cardiac tissue on day 41.

SUPPLEMENTARY VIDEO 2 AAV6-shGJA1-treated hiPSC-derived cardiac tissue on day 41.

SUPPLEMENTARY VIDEO 3 Calcium imaging of AAV6-scramble shRNA-treated hiPSC-CMs.

SUPPLEMENTARY VIDEO 4 Calcium imaging of AAV6-shGJA1-treated hiPSC-CMs.

## Abbreviations

AAV, adeno-associated virus; Cx43, Connexin 43; hiPSC-CMs, human-induced pluripotent stem cell-derived cardiomyocytes.

## References

[B1] Angelis N. Baulies A. Hubl F. Kucharska A. Kelly G. Llorian M. (2024). Loss of ARID3A perturbs intestinal epithelial proliferation-differentiation ratio and regeneration. J. Exp. Med. 221, e20232279. 10.1084/jem.20232279 39150450 PMC11329776

[B2] Belmont P. J. Chen W. J. San Pedro M. N. Thuerauf D. J. Gellings Lowe N. Gude N. (2010). Roles for endoplasmic reticulum-associated degradation and the novel endoplasmic reticulum stress response gene Derlin-3 in the ischemic heart. Circ. Res. 106, 307–316. 10.1161/circresaha.109.203901 19940266 PMC3330119

[B4] Besser R. R. Ishahak M. Mayo V. Carbonero D. Claure I. Agarwal A. (2018). Engineered microenvironments for maturation of stem cell derived cardiac myocytes. Theranostics 8, 124–140. 10.7150/thno.19441 29290797 PMC5743464

[B5] Chacar S. Abdrabou W. Al Hageh C. Ali L. Venkatachalam T. Zalloua P. (2025). Remodeling of the cardiac striatin interactome and its dynamics in the diabetic heart. Sci. Rep. 15, 7384. 10.1038/s41598-025-91098-6 40025125 PMC11873221

[B6] Chang Q. Zhao S. Sun J. Guo W. Yang L. Qiu L. (2025). Identification of a novel prognostic and therapeutic prediction model in clear cell renal carcinoma based on Renin-angiotensin system related genes. Front. Endocrinol. (Lausanne) 16, 1521940. 10.3389/fendo.2025.1521940 40099255 PMC11911175

[B7] Chen L. (2022). TAOK1 promotes proliferation and invasion of non-small-cell lung cancer cells by inhibition of WWC1. Comput. Math. Methods Med. 2022, 1–9. 10.1155/2022/3157448 PMC9499761 36158126

[B8] Chen X. Sun Z. Zhou S. Jiang W. Li J. Song G. (2023). SH3 domain‐binding kinase 1 promotes proliferation and inhibits apoptosis of cervical cancer via activating the Wnt/β‐catenin and Raf/ERK1/2 signaling pathways. Mol. Carcinog. 62, 1147–1162. 10.1002/mc.23552 37132991

[B9] Diguet N. Trammell S. A. J. Tannous C. Deloux R. Piquereau J. Mougenot N. (2018). Nicotinamide riboside preserves cardiac function in a mouse model of dilated cardiomyopathy. Circulation 137, 2256–2273. 10.1161/circulationaha.116.026099 29217642 PMC6954688

[B10] Du L. Liu N. Jin J. Cao M. Sun Y. Gao X. (2022). ZNF3 regulates proliferation, migration and invasion through MMP1 and TWIST in colorectal cancer. Acta Biochim. Biophys. Sin. (Shanghai). 54, 1889–1896. 10.3724/abbs.2022187 36789689 PMC10157515

[B12] Fontes M. S. van Veen T. A. de Bakker J. M. van Rijen H. V. (2012). Functional consequences of abnormal Cx43 expression in the heart. Biochim. Biophys. Acta 1818, 2020–2029. 10.1016/j.bbamem.2011.07.039 21839722

[B13] Gao G. Li X. Wu H. Huang L. L. Lin Y. X. Huo Z. (2023). LncRNA SNHG6 upregulates KPNA5 to overcome gemcitabine resistance in pancreatic cancer via sponging miR-944. Pharm. (Basel) 16, 184. 10.3390/ph16020184 PMC9961296 37259332

[B14] Graham S. V. Jiang J. X. Mesnil M. (2018). Connexins and pannexins: important players in tumorigenesis, metastasis and potential therapeutics. Int. J. Mol. Sci. 19, 1645. 10.3390/ijms19061645 29865195 PMC6032133

[B15] Gutstein D. E. Morley G. E. Tamaddon H. Vaidya D. Schneider M. D. Chen J. (2001). Conduction slowing and sudden arrhythmic death in mice with cardiac-restricted inactivation of connexin43. Circ. Res. 88, 333–339. 10.1161/01.res.88.3.333 11179202 PMC3630465

[B17] Hasenfuss G. Mulieri L. A. Blanchard E. M. Holubarsch C. Leavitt B. J. Ittleman F. (1991). Energetics of isometric force development in control and volume-overload human myocardium. Comparison with animal species. Circ. Res. 68, 836–846. 10.1161/01.res.68.3.836 1742869

[B19] Hinata Y. Sasaki D. Matsuura K. Shimizu T. (2024). Induction of cardiac alternans in human iPS-derived cardiomyocytes through beta-adrenergic receptor stimulation. Physiol. Rep. 12, e70152. 10.14814/phy2.70152 39715724 PMC11666346

[B20] Hino H. Dai P. Yoshida T. Hatakeyama T. Harada Y. Otsuji E. (2015). Interaction of Cx43 with Hsc70 regulates G1/S transition through CDK inhibitor p27. Sci. Rep. 5, 15365. 10.1038/srep15365 26481195 PMC4612729

[B22] Hou M. Liu S. Yan K. Sun Z. Li S. (2023). Downregulation of odontogenic ameloblast-associated protein in the progression of periodontal disease affects cell adhesion, proliferation, and migration. Arch. Oral Biol. 145, 105588. 10.1016/j.archoralbio.2022.105588 36442302

[B23] Hua G. He C. Lv X. Fan L. Wang C. Remmenga S. W. (2016). The four and a half LIM domains 2 (FHL2) regulates ovarian granulosa cell tumor progression via controlling AKT1 transcription. Cell Death Dis. 7, e2297. 10.1038/cddis.2016.207 27415427 PMC4973349

[B24] Ionta M. Ferreira R. A. Pfister S. C. Machado-Santelli G. M. (2009). Exogenous Cx43 expression decrease cell proliferation rate in rat hepatocarcinoma cells independently of functional gap junction. Cancer Cell Int. 9, 22. 10.1186/1475-2867-9-22 19678939 PMC2738655

[B25] Jiang M. Song Y. Chen X. Lu W. Zhu M. Wei M. (2023). COX6A2 deficiency leads to cardiac remodeling in human pluripotent stem cell-derived cardiomyocytes. Stem Cell Res. Ther. 14, 357. 10.1186/s13287-023-03596-x 38072986 PMC10712066

[B26] Karbassi E. Fenix A. Marchiano S. Muraoka N. Nakamura K. Yang X. (2020). Cardiomyocyte maturation: advances in knowledge and implications for regenerative medicine. Nat. Rev. Cardiol. 17, 341–359. 10.1038/s41569-019-0331-x 32015528 PMC7239749

[B27] Kim J. K. Lee S. Y. Park C. W. Park S. H. Yin J. Kim J. (2014). Rab3a promotes brain tumor initiation and progression. Mol. Biol. Rep. 41, 5903–5911. 10.1007/s11033-014-3465-2 24965146

[B28] Kiss E. Fischer C. Sauter J. M. Sun J. Ullrich N. D. (2022). The structural and the functional aspects of intercellular communication in iPSC-Cardiomyocytes. Int. J. Mol. Sci. 23, 4460. 10.3390/ijms23084460 35457277 PMC9031673

[B30] Kodama M. Furutani K. Kimura R. Ando T. Sakamoto K. Nagamori S. (2019). Systematic expression analysis of genes related to generation of action potentials in human iPS cell-derived cardiomyocytes. J. Pharmacol. Sci. 140, 325–330. 10.1016/j.jphs.2019.06.006 31279582

[B31] Kong X. Wang J. S. Yang H. (2021). Upregulation of lncRNA DARS-AS1 accelerates tumor malignancy in cervical cancer by activating cGMP-PKG pathway. J. Biochem. Mol. Toxicol. 35, 1–11. 10.1002/jbt.22749 33634536

[B32] Kostin S. Rieger M. Dammer S. Hein S. Richter M. Klovekorn W. P. (2003). Gap junction remodeling and altered connexin43 expression in the failing human heart. Mol. Cell Biochem. 242, 135–144. 10.1023/a:1021154115673 12619876

[B33] Kucera J. P. Prudat Y. Marcu I. C. Azzarito M. Ullrich N. D. (2015). Slow conduction in mixed cultured strands of primary ventricular cells and stem cell-derived cardiomyocytes. Front. Cell Dev. Biol. 3, 58. 10.3389/fcell.2015.00058 26442264 PMC4585316

[B34] Kurotsu S. Sadahiro T. Fujita R. Tani H. Yamakawa H. Tamura F. (2020). Soft matrix promotes cardiac reprogramming via inhibition of YAP/TAZ and suppression of fibroblast signatures. Stem Cell Rep. 15, 612–628. 10.1016/j.stemcr.2020.07.022 PMC7486305 32857980

[B68] Liu H. Zhou R. Li S. Dong J. Fang Y. Luo Y. (2024). Epigenetic repression 1169 of Cend1 by lysine-specific demethylase 1 is essential for murine heart development. 1170 iScience 27, 108722. 10.1016/j.isci.2023.108722 PMC10788269 38226173

[B37] Lu J. Wang Y. H. Huang X. Y. Xie J. W. Wang J. B. Lin J. X. (2020). circ-CEP85L suppresses the proliferation and invasion of gastric cancer by regulating NFKBIA expression via miR-942-5p. J. Cell Physiol. 235, 6287–6299. 10.1002/jcp.29556 32026471

[B38] Marcu I. C. Illaste A. Heuking P. Jaconi M. E. Ullrich N. D. (2015). Functional characterization and comparison of intercellular communication in stem cell-derived cardiomyocytes. Stem Cells 33, 2208–2218. 10.1002/stem.2009 25968594

[B39] Mashali M. A. Saad N. S. Canan B. D. Elnakish M. T. Milani-Nejad N. Chung J. H. (2021). Impact of etiology on force and kinetics of left ventricular end-stage failing human myocardium. J. Mol. Cell Cardiol. 156, 7–19. 10.1016/j.yjmcc.2021.03.007 33766524 PMC8217133

[B40] Matsuura K. Seta H. Haraguchi Y. Alsayegh K. Sekine H. Shimizu T. (2016). TRPV-1-mediated elimination of residual iPS cells in bioengineered cardiac cell sheet tissues. Sci. Rep. 6, 21747. 10.1038/srep21747 26888607 PMC4757885

[B41] Matsuura K. Wada M. Shimizu T. Haraguchi Y. Sato F. Sugiyama K. (2012). Creation of human cardiac cell sheets using pluripotent stem cells. Biochem. Biophys. Res. Commun. 425, 321–327. 10.1016/j.bbrc.2012.07.089 22842572

[B42] Mehta P. P. Perez-Stable C. Nadji M. Mian M. Asotra K. Roos B. A. (1999). Suppression of human prostate cancer cell growth by forced expression of connexin genes. Dev. Genet. 24, 91–110. 10.1002/(sici)1520-6408(1999)24:1/2<91::aid-dvg10>3.0.co;2-# 10079514

[B43] Nakamura S. Numata G. Yamaguchi T. Tokiwa H. Higashikuni Y. Nomura S. (2022). Endoplasmic reticulum stress-activated nuclear factor-kappa B signaling pathway induces the upregulation of cardiomyocyte dopamine D1 receptor in heart failure. Biochem. Biophys. Res. Commun. 637, 247–253. 10.1016/j.bbrc.2022.11.031 36410273

[B44] Ogawa M. Geng F. S. Humphreys D. T. Kristianto E. Sheng D. Z. Hui S. P. (2021). Kruppel-like factor 1 is a core cardiomyogenic trigger in zebrafish. Science 372, 201–205. 10.1126/science.abe2762 33833125

[B46] Porrello E. R. Mahmoud A. I. Simpson E. Hill J. A. Richardson J. A. Olson E. N. (2011). Transient regenerative potential of the neonatal mouse heart. Science 331, 1078–1080. 10.1126/science.1200708 21350179 PMC3099478

[B47] Reaume A. G. de Sousa P. A. Kulkarni S. Langille B. L. Zhu D. Davies T. C. (1995). Cardiac malformation in neonatal mice lacking connexin43. Science 267, 1831–1834. 10.1126/science.7892609 7892609

[B48] Ronaldson-Bouchard K. Ma S. P. Yeager K. Chen T. Song L. Sirabella D. (2018). Advanced maturation of human cardiac tissue grown from pluripotent stem cells. Nature 556, 239–243. 10.1038/s41586-018-0016-3 29618819 PMC5895513

[B49] Sasaki D. Matsuura K. Seta H. Haraguchi Y. Okano T. Shimizu T. (2018). Contractile force measurement of human induced pluripotent stem cell-derived cardiac cell sheet-tissue. PLoS One 13, e0198026. 10.1371/journal.pone.0198026 29791489 PMC5965888

[B50] Shen M. Chen Y. Tang W. Ming M. Tian Y. Ding F. (2022). Semaphorin 3E promote Schwann cell proliferation and migration. Exp. Cell Res. 412, 113019. 10.1016/j.yexcr.2022.113019 35085549

[B51] Shibata M. Kanda M. Tanaka H. Umeda S. Miwa T. Shimizu D. (2017). Overexpression of Derlin 3 is associated with malignant phenotype of breast cancer cells. Oncol. Rep. 38, 1760–1766. 10.3892/or.2017.5800 28713959

[B52] Sottas V. Wahl C. M. Trache M. C. Bartolf-Kopp M. Cambridge S. Hecker M. (2018). Improving electrical properties of iPSC-cardiomyocytes by enhancing Cx43 expression. J. Mol. Cell Cardiol. 120, 31–41. 10.1016/j.yjmcc.2018.05.010 29777691

[B53] Takada T. Sasaki D. Matsuura K. Miura K. Sakamoto S. Goto H. (2022). Aligned human induced pluripotent stem cell-derived cardiac tissue improves contractile properties through promoting unidirectional and synchronous cardiomyocyte contraction. Biomaterials 281, 121351. 10.1016/j.biomaterials.2021.121351 34979417

[B55] Takahashi K. Yamanaka S. (2006). Induction of pluripotent stem cells from mouse embryonic and adult fibroblast cultures by defined factors. Cell 126, 663–676. 10.1016/j.cell.2006.07.024 16904174

[B56] Tarasov K. V. Chakir K. Riordon D. R. Lyashkov A. E. Ahmet I. Perino M. G. (2022). A remarkable adaptive paradigm of heart performance and protection emerges in response to marked cardiac-specific overexpression of ADCY8. Elife 11, e80949. 10.7554/elife.80949 36515265 PMC9822292

[B57] van Kempen M. J. ten Velde I. Wessels A. Oosthoek P. W. Gros D. Jongsma H. J. (1995). Differential connexin distribution accommodates cardiac function in different species. Microsc. Res. Tech. 31, 420–436. 10.1002/jemt.1070310511 8534903

[B58] Vermij S. H. Abriel H. van Veen T. A. (2017). Refining the molecular organization of the cardiac intercalated disc. Cardiovasc Res. 113, 259–275. 10.1093/cvr/cvw259 28069669

[B59] Wang H. Chen Y. Yuan Q. Chen L. Dai P. Li X. (2022). HRK inhibits colorectal cancer cells proliferation by suppressing the PI3K/AKT/mTOR pathway. Front. Oncol. 12, 1053510. 10.3389/fonc.2022.1053510 36568155 PMC9769574

[B60] Wang H. Feng Y. Bao Z. Jiang C. Yan W. Wang Y. (2013). Epigenetic silencing of KAZALD1 confers a better prognosis and is associated with malignant transformation/progression in glioma. Oncol. Rep. 30, 2089–2096. 10.3892/or.2013.2706 24002581

[B61] Wang R. Chen X. Xu T. Xia R. Han L. Chen W. (2016). MiR-326 regulates cell proliferation and migration in lung cancer by targeting phox2a and is regulated by HOTAIR. Am. J. Cancer Res. 6, 173–186. 27186394 PMC4859651

[B62] Wang Y. Li Q. Tao B. Angelini M. Ramadoss S. Sun B. (2023). Fibroblasts in heart scar tissue directly regulate cardiac excitability and arrhythmogenesis. Science 381, 1480–1487. 10.1126/science.adh9925 37769108 PMC10768850

[B64] Wu A. Zhou X. Mi L. Shen J. (2020). LINC00202 promotes retinoblastoma progression by regulating cell proliferation, apoptosis, and aerobic glycolysis through miR-204-5p/HMGCR axis. Open Life Sci. 15, 437–448. 10.1515/biol-2020-0047 33817232 PMC7874641

[B65] Wyant G. A. Jiang Q. Singh M. Qayyum S. Levrero C. Maron B. A. (2024). Induction of DEPP1 by HIF mediates multiple hallmarks of ischemic cardiomyopathy. Circulation 150, 770–786. 10.1161/circulationaha.123.066628 38881449 PMC11361356

[B66] Xu F. Yu Y. Wang F. Sun W. Li P. Wu H. F. (2020). Analysis of gene expression profiling of amyloidogenic immunoglobulin light-chains on cultured rat cardiomyocytes. Exp. Ther. Med. 19, 3767–3777. 10.3892/etm.2020.8610 32346441 PMC7185198

[B67] Yuan X. Braun T. (2017). Multimodal regulation of cardiac myocyte proliferation. Circ. Res. 121, 293–309. 10.1161/circresaha.117.308428 28729454

