# Supplementary material for: Connexin 43 suppression enhances contractile force in human iPSC-derived cardiac tissues
Source: Front Bioeng Biotechnol. 2025 Aug 8;13:1615953. doi: 10.3389/fbioe.2025.1615953 (PMC12371756; doi:10.3389/fbioe.2025.1615953)
Supplement: Supplementary file 2 [file Supplementaryfile1.docx]

**Supplementary Materials 1**

**Materials and methods**

***AAV serotype 6-mediated regulation of the GJA1 gene in hiPSC-derived cardiac tissue***

We employed an AAV serotype 6 vector to mediate *GJA1* overexpression (OE), knockdown (shGJA1), and GCaMP expression in bioengineered hiPSC-derived cardiac tissues. Viral particles containing the AAV serotype 6 vector were constructed to express *GJA1* and EGFP genes linked by a T2A sequence (AAV-GJA1-T2A-GFP), or EGFP alone, driven by the cytomegalovirus (CMV) promoter. The following vectors were constructed and packaged by VectorBuilder (Santa Clara, CA): pAAV[Exp]-CMV>EGFP:WPRE (VB010000-9394npt), pAAV[Exp]-CMV>hGJA1[NM_000165.5](ns):T2A:EGFP: WPRE (VB900089-0611jqt), pAAV[shRNA]-EGFP-U6>Scramble_shRNA (VB010000-0023jze), pAAV[shRNA]-EGFP-U6>hGJA1[shRNA#1] (VB900137-4392ndg), and pAAV[Exp]-CMV>GCaMP6f:WPRE (VB220427-1461cuw). Vector IDs can be used to retrieve additional information about these vectors on vectorbuilder.com. To assess potential off-target effects of the shRNA construct targeting GJA1, we performed a nucleotide BLAST search (blastn) using the shRNA sense strand sequence (GGTGGTAATTGTGGCTAAATACTCGAGTATTTAGCCACAATTACCACC) against the human transcriptome database [Human G+T, somewhat similar sequences (blastn), word size: 7, E-value: 100, match/mismatch scores: 1/-3, filtering: none]. Among the 93 transcript hits identified, the 92 transcript was not observed with any differentially expressed genes (DEGs) identified in our RNA-seq data, except for the intended target GJA1, indicating minimal risk of off-target interactions.

***Motion capture analysis of hiPSC-CMs on fibrin gel***

Motion capture analysis was performed in accordance with the instrument manual and our previous studies (Takada T et al., 2022; Takahashi H et al., 2018). In brief, microscopic videos of the cardiac tissues were recorded using an inverted microscope equipped with a CMOS color camera (TOSHIBA, Tokyo, Japan). Subsequently, we set the ROI (region of Interest) to the individual cardiomyocytes in the tissue. In the other words, we evaluated the contractile timing of each cellular level region in the tissue. Thus, the moving distance to X-axis of the ROI (cardiomyocyte). The movement along the X-axis of cardiomyocytes and the time course at designated points, including the four corners and the center of the cardiac tissue, were analyzed using the motion analysis tool ViewPoint (Glenallan Technology Inc., Clinton, NY, USA).

The time from the start of measurement to the first peak contraction along the X-axis at five designated points within the cardiac tissues was extracted. Finally, the standard deviation of the times was calculated. When the standard deviation is smaller, the ROI (cardiomyocyte) moves with more synchronous timing at each point. It indicates that the cardiomyocytes in the cardiac tissues perform the synchronous contraction with each other. A smaller standard deviation indicates that cardiomyocytes are contracting with more synchronous timing across the measured points, as noted in previous studies (Takada T et al., 2022). The standard deviation of these times was compared between control and *GJA1*-OE cardiac tissues, as well as between scramble and sh*GJA1* cardiac tissues. For the analysis, the video files were converted into static images, resulting in a compression of the actual time by one-third.

***RNA extraction, quantitative real-time PCR, and RNA sequencing***

Total RNA was extracted from cells on day 44 using the RNeasy Mini Kit (Qiagen, Hilden, Germany), following the manufacturer’s protocol. RNA concentration and quality were assessed using a Nanodrop ND-1000 spectrophotometer (Thermo Fisher Scientific). First-strand cDNA was synthesized from the purified total RNA using the High Capacity cDNA Reverse Transcription Kit (Thermo Fisher Scientific) on the Applied Biosystems ProFlex™ PCR System (Thermo Fisher Scientific). Quantitative real-time PCR was conducted on an Applied Biosystems® ViiA™ 7 real-time PCR system (Thermo Fisher Scientific), in accordance with the manufacturer's guidelines. Gene expression levels were analyzed using TaqMan gene expression assays (Thermo Fisher Scientific) with primers targeting *GAPDH*, *GJA5*, *GJC1*, *MYL2*, *MYH7*, *RYR2*, *ATP2A2*, *PLN*, *CACNA1C*, *SCN5A*, *KCNH2*, *KCNJ2*, *TNNI3*, *HCN4*, *ERBB1*, *ERBB4*, and *NPPB* (**Supplementary Table**). Data were evaluated using the △CT method, with statistical analyses based on △CT comparisons between groups, and results presented as fold change ± SD (2^−△CT^). We considered it was important to evaluate GJA1 expression levels in native human heart tissue and compare them with those observed in hiPSC-CMs to better validate and interpret our findings. For this comparison, we used commercially available pooled RNA from normal adult human heart tissue (pooled from three male Caucasian donors, aged 30–39; Human Heart Total RNA, Catalog No. 636532; lot 2002947A, Takara Bio USA, Inc.). The relative expression values were calculated by setting the expression level in adult human heart tissue to 1 and comparing the gene expression in the four experimental groups (control, GJA1-OE, scramble, and shGJA1; each group n = 7). We included the 95% confidence interval (CI) to indicate the precision and variability of the gene expression measurements relative to the adult heart sample. If the 95% CI crosses 1, it suggests that the expression level is not significantly different from that in the adult heart. The 95% CIs for each group were calculated from the mean, standard deviation, and sample size (n = 7) of their relative expression levels normalized to adult heart tissue. Since we only had one pooled adult heart sample (i.e., not multiple biological replicates), we used 95% CI as an approximate reference to evaluate potential differences, acknowledging its limitations. RNA sequencing and gene ontology analyses (n = 3) were outsourced to Rhelixa (Tokyo, Japan). They used Wald test after RLE normalization with DEseq2 based on a previous report (Love MI et al., 2014).

***Western blotting***

For western blotting, hiPSC-derived cardiac tissues were dissected and homogenized using a homogenizer pestle (AS ONE, Osaka, Japan) and the BIORUPTOR (COSMO BIO, Tokyo, Japan) in RIPA Lysis Buffer containing 2 mM PMSF in DMSO, a protease inhibitor cocktail in DMSO, and 1 mM sodium orthovanadate in water, following the manufacturer’s instructions (Santa Cruz Biotechnology). Protein concentrations of the tissue lysates were determined by creating a standard curve with BSA standard solution and measuring the absorbance at 562 nm using a bicinchoninic acid (BCA) protein assay kit (Takara Bio Inc., Shiga, Japan) and a microplate reader system (SpectraMax M2e and SoftMax^®︎^ Pro software, Molecular Devices, CA, USA).

After determining protein concentrations, the samples were heated to 95 °C for 10 min in 4x Laemmli sample buffer (277.8 mM Tris, 4.4% LDS, 44.4% glycerol, 0.02% bromophenol blue, pH 6.8; Bio-Rad, CA, USA) containing 2-mercaptoethanol. Equal amounts of protein were then loaded onto Criterion™ TGX Stain-Free™ 4–15% or Any kD precast polyacrylamide gels (Bio-Rad) and transferred to low-autofluorescence PVDF membranes using the Transfer Kit and Trans-Blot Turbo Transfer System (Bio-Rad). Stain-Free technology (Bio-Rad) was employed to confirm equal protein loading.

Membranes were blocked in EveryBlot Blocking Buffer (Bio-Rad) for 5 min at room temperature and incubated overnight at 4°C with primary antibodies. The primary antibody for Cx43 (1:2000, Abcam ab11370) was used to detect protein levels. Following membrane washes with PBS-T the next day, membranes were incubated with appropriate HRP-conjugated secondary antibodies (1:10,000 goat anti-rabbit IgG H&L (HRP), Abcam) and visualized using Clarity Western ECL Substrate (Bio-Rad). Chemiluminescence signals were captured using a ChemiDoc Imaging System (Bio-Rad), and band density was calculated using ImageLab™ software (Version 6.0.1, build 34, Bio-Rad). Each band's density was normalized to the total protein loaded in each lane, measured with the Bio-Rad Stain-Free Gel System, and statistically compared.

***Immunofluorescent staining***

The hiPSC-derived cardiac tissues were fixed in 4% paraformaldehyde for 15 min, permeabilized with 0.15% Triton X-100 in PBS, and blocked with 2% BSA in PBS for 20 min at room temperature. The tissues were then incubated with a mouse monoclonal anti-cTnT antibody (1:200, Thermo Fisher Scientific) and a rabbit polyclonal anti-Cx43 antibody (1:300, Abcam). After primary antibody incubation, the samples were treated with Alexa Fluor 568-conjugated anti-mouse IgG (H+L) (1:200, Thermo Fisher Scientific) and Alexa Fluor 647-conjugated anti-rabbit IgG (H+L) (1:200, Thermo Fisher Scientific). Following staining, the tissues were mounted on coverslips with an anti-fade solution (ProLong Gold Antifade Reagent with DAPI, Thermo Fisher Scientific). Confocal fluorescence imaging was performed using a FV1200 microscope (Olympus, Tokyo, Japan).

***Corrected Field Potential Duration (cFPD)***

Extracellular Field Potential (EFP) recordings of AAV6-treated hiPSC-CMs were obtained using the CardioExcyte 96 system (Nanion Technologies, Munich, Germany). Nanion CardioExcyte 96 Sensor Plates were first coated with medium A containing 20 μg/mL fibronectin (Corning) and 2.5 mg/mL aminocaproic acid (Sigma-Aldrich) for over 2 h at 37°C. On day 29, 1.1 × 10⁵ hiPSC-CMs, seeded at the same density as the cells on the fibrin gel, were placed into each well. The protocol for AAV6 transduction and subsequent medium changes followed the same methods described in the main manuscript.

On day 44, EFP measurements were taken using the CardioExcyte 96 system and CardioExcyte Control software (Nanion Technologies). Video recordings of the EFP were captured every 5 min for over 2 h. Following this, 10 μM of isoproterenol was added to each well, and EFP was measured again for an additional 2 h. The data analysis on calculating field potential duration (FPD) from the EFP data was performed by Nanion Technologies using DataControl 96 software (Nanion). The FPD was then corrected using Fridericia's formula, in accordance with the manufacturer’s guidelines and previous research (Wei X et al., 2022; Benkel T et al., 2022).

***Simultaneous intracellular calcium imaging***

On day 29, 1.1 × 10⁵ hiPSC-CMs were seeded into 96-well plates that had been pre-coated with medium A containing 20 μg/mL fibronectin (Corning) and 2.5 mg/mL aminocaproic acid (Sigma-Aldrich) for over 2 h at 37 °C. Following the AAV6 transduction protocol mentioned above, an additional AAV6 was used to introduce GCaMP6f into the hiPSC-CMs. The MOI was set at 1.5 × 10⁴ genome copies. On day 42, simultaneous intracellular calcium imaging of hiPSC-CMs treated with either AAV6-scramble shRNA or shGJA1 was captured using a BZ-X800 microscope (KEYENCE, IL, USA). The images were analyzed by Spiky: An ImageJ Plugin (Pasqualin C et al., 2022).

**Supplementary Table. PCR primer information**

| **Gene name** | **Thermo Fisher Scientific No.** |
| --- | --- |
| glyceraldehyde-3-phosphate dehydrogenase (GAPDH) | Hs99999905_m1 |
| myosin, light chain 2, regulatory, cardiac, slow (MYL2) | Hs00166405_m1 |
| myosin, heavy chain 7, cardiac, muscle, beta (MYH7) | Hs01110632_m1 |
| ATPase, Ca++ transporting, cardiac muscle, slow twitch 2 (ATP2A2) | Hs00544877_m1 |
| ryanodine receptor 2 (cardiac) (RYR2) | Hs00181461_m1 |
| gap junction protein, alpha 1, 43kDa (GJA1) | Hs00748445_s1 |
| gap junction protein alpha 5 (GJA5) | Hs00270952_s1 |
| gap junction protein gamma 1 (GJC1) | Hs00271416_s1 |
| phospholamban (PLN) | Hs00160179_m1 |
| calcium channel, voltage-dependent, L type, alpha 1C subunit (CACNA1C) | Hs00167681_m1 |
| sodium channel, voltage-gated, type V, alpha subunit (SCN5A) | Hs00165693_m1 |
| potassium voltage-gated channel, subfamily H (eag-related), member 2 (KCNH2) | Hs00165120_m1 |
| potassium inwardly-rectifying channel, subfamily J, member 2 (KCNJ2) | Hs00265315_m1 |
| troponin I3, cardiac type (TNNI3) | Hs00165957_m1 |
| hyperpolarization activated cyclic nucleotide gated potassium channel 4 (HCN4) | Hs00175760_m1 |
| epidermal growth factor receptor (EGFR; ERBB1) | Hs01076090_m1 |
| erb-b2 receptor tyrosine kinase 4 (ERBB4) | Hs00955525_m1 |
| natriuretic peptide B (NPPB) | Hs01057466_g1 |

PCR; polymerase chain reaction

**Supplementary Figure 1. Confocal microscopic images of AAV6-GFP, GJA1-OE, scramble, and shGJA1 treated hiPSC-derived cardiac tissues.**

Scale bar = 100 μm

The yellow arrow indicates that Cx43 is aggregated in AAV6-GJA1-OE cardiac tissue, whereas the white arrow marks Cx43 in AAV6-shGJA1 tissue.

AAV = adeno-associated virus; cTnT = cardiac troponin T; hiPSC = human induced pluripotent stem cells; OE = overexpression

**Supplementary Figure 2. Gene expression levels in AAV6-GFP and GJA1 treated hiPSC-CMs**

**Each dot represents an experiment (n > 4 for each group).**

AAV = adeno-associated virus; hiPSC-CMs = human induced pluripotent stem cell-derived cardiomyocytes; OE = overexpression

**Supplementary Figure 3.** **Relative expression levels of GJA1 gene normalized to the levels of adult heart**

GJA1 gene expression levels for each data were normalized to the levels of adult (mean with 95% confidence interval). Adult: normal human adult heart.

Each dot represents an experiment (n = 7 for each group except for the adult group).

OE = overexpression

**Supplementary Figure 4. Characteristics and functions of AAV6-GJA1-OE cardiac tissues**

A: Contractile and relaxation function in AAV6-GFP and GJA1 treated hiPSC-derived cardiac tissues at spontaneous beating (n = 10). B: Force-frequency relationship between AAV6-GFP and GJA1 treated hiPSC-derived cardiac tissues. C: Contractile and relaxation function after 1μM of ISP treatment in AAV6-GFP and GJA1 treated hiPSC-derived cardiac tissues (n = 10). D: Contractile and relaxation function at 100 ppm after 1μM of ISP treatment in AAV6-GFP and GJA1 treated hiPSC-derived cardiac tissues (n = 10). E: Corrected field potentials of AAV6-GFP and GJA1 treated hiPSC-CMs before (n = 8 and n = 6, respectively). and after ISP treatment (n = 8 and n = 5, respectively). Each dot represents data from a single well in 96-well plates respectively, based on 4 independent experiments.

AAV = adeno-associated virus; hiPSC-CMs = human induced pluripotent stem cell-derived cardiomyocytes; ISP = isoproterenol; OE = overexpression; ppm = pacing per minute.

**Supplementary Figure 5. Gene expression levels in AAV6-scramble shRNA and shGJA1 treated hiPSC-CMs**

Each dot represents an experiment (n > 3 for each group).

AAV = adeno-associated virus; hiPSC-CMs = human induced pluripotent stem cell-derived cardiomyocytes

**Supplementary Figure 6. Simultaneous intracellular calcium imaging of AAV6-scramble shRNA- and shGJA1 treated hiPSC-CMs**

(n = 1)

AAV = adeno-associated virus; hiPSC-CMs = human induced pluripotent stem cell-derived cardiomyocytes

**Supplementary Figure 7. Characteristics and functions of AAV6-shGJA1 cardiac tissues**

A: Contractile and relaxation function in AAV6-Scramble and shGJA1 treated cardiac tissues at spontaneous beating (n = 7). B: Force-frequency relationship between AAV6-Scramble and shGJA1 treated cardiac tissues. More than three experiments were conducted for each pacing frequency (n > 3). C: Contractile and relaxation function in AAV6-Scramble and shGJA1 treated cardiac tissues after 1μM of ISP treatment (n = 7). D: Contractile and relaxation function in AAV6-Scramble and shGJA1 treated cardiac tissues at 100 ppm after 1μM of ISP treatment (n = 7). E: Corrected field potentials of AAV6-Scramble and shGJA1 treated hiPSC-CMs before (n = 7) and after ISP treatment (n = 4 and n = 6, respectively). Each dot represents data from a single well in 96-well plates respectively, based on 3 independent experiments.

AAV = adeno-associated virus; bpm = beating per minute; hiPSC = human induced pluripotent stem cells; ISP = isoproterenol; ppm = pacing per minute

**Supplementary Figure 8. Heat map involved control vs GJA1 and scramble vs shGJA1**

(n = 3)

OE = overexpression

**Supplementary Figure 9. Gene ontology analyses**

FC = fold change

**Supplementary Figure 10. Downstream effects of GJA1 gene regulation**


(A) Common genes identified between the downregulation of GJA1-OE compared to control and upregulation of shGJA1 compared to scramble (n = 3). (B) Biological process terms of 19 common genes

OE = overexpression

**Supplementary Figure 11. Log₂ fold changes of 19 common DEGs in shGJA1 and GJA1-OE, relative to scramble and control, respectively**

N = 3. Error bar indicates standard error. DEG = differentially expressed gene. OE = overexpression

**Supplementary Figure 12. Images of unprocessed blots used for Figure 2B and 3B**

**References**

Benkel T, Zimmermann M, Zeiner J, Bravo S, Merten N, Lim VJY, et al. (2022). How Carvedilol activates beta(2)-adrenoceptors. Nat Commun. 13, 7109.

Love MI, Huber W, Anders S. (2014). Moderated estimation of fold change and dispersion for RNA-seq data with DESeq2. Genome Biol. 15, 550.

Pasqualin C, Gannier F, Yu A, Benoist D, Findlay I, Bordy R, et al. (2022). Spiky: An ImageJ Plugin for Data Analysis of Functional Cardiac and Cardiomyocyte Studies. J Imaging. 8.

Takada T, Sasaki D, Matsuura K, Miura K, Sakamoto S, Goto H, et al. (2022). Aligned human induced pluripotent stem cell-derived cardiac tissue improves contractile properties through promoting unidirectional and synchronous cardiomyocyte contraction. Biomaterials. 281, 121351.

Takahashi H, Shimizu T, Okano T. (2018). Engineered Human Contractile Myofiber Sheets as a Platform for Studies of Skeletal Muscle Physiology. Sci Rep. 8, 13932.

Wei X, Chang ACH, Chang H, Xu S, Xue Y, Zhang Y, et al. (2022). Hypoglycemia-Exacerbated Mitochondrial Connexin 43 Accumulation Aggravates Cardiac Dysfunction in Diabetic Cardiomyopathy. Front Cardiovasc Med. 9, 800185.
